# The value of arthroplasty registry data

**DOI:** 10.3109/17453671003667184

**Published:** 2010-03-31

**Authors:** Stephen E Graves

**Affiliations:** AOA National Joint Replacement Registry, Data Management and Analysis Centre, University of Adelaide, SAAustralia

## Introduction

Arthroplasty registries play a critical role in improving the outcome of joint replacement surgery. They provide unique community-based comparative data that enables individual surgeons to identify best practice that is relevant to their own approach to arthroplasty surgery. Registries simultaneously compare the effect of multiple factors on the outcome of joint replacement, and through ongoing monitoring are also sensitive to the impact of changing practice. The information they provide is known to change practice in a beneficial manner. There continues, however, to be an ongoing debate about the value of registry data.

Traditionally, the value of clinical information has been determined by its standing in the hierarchy of levels of clinical evidence. The underlying basis for this hierarchy is the ability of the data to establish causality with respect to outcome. This has been specifically linked to study design, with a randomized controlled trial (RCT) being recognized as having the greatest capacity to achieve this. If the clinical evidence approach is used to categorize registry-derived data, there is no option other than to regard this information as coming from an observational study. As such, registry data would be considered as having lower value than an RCT or a systematic review of RCTs. This could be correct with respect to ability to establish causality. However, is this clinically relevant and is it the best approach to determine comparative value of information obtained from registries and clinical trials?

Clinical trials are designed to provide evidence to prove a hypothesis. Important in their design is the need to limit from the outset the number of confounding factors that may have an impact on data analysis and its subsequent interpretation. Study design predetermines where the trial will be undertaken, the surgeons involved, and which patients will be included, as well as the surgical technique and the prostheses to be used. Critical to the design is ensuring that the trial is adequately powered to enable statistical difference for the relevant parameter(s) to be compared. A trial has an end. When a trial is designed, it is necessary to make assumptions. The accuracy and relevance of those assumptions will affect the study design and will depend on available knowledge and the understanding of that knowledge by the designers of the trial.

A registry is not a clinical trial, and establishing causality is not its focus. A registry is an ongoing quality assurance mechanism that is designed to identify and monitor differences in comparative outcomes within the community being surveyed. The community may be a specific region, an entire country, or even a number of countries combined. As registries have a different purpose, their approach to data collection and analysis is entirely different from a clinical trial. They attempt to ensure that there are no exclusions. All hospitals, surgeons, patients, surgical techniques, and prostheses are included. Analyses are undertaken to identify different outcomes associated with these and other factors. The analysis is reported for a defined period. A registry is ongoing, and as such is able to monitor changing practice and the impact of that change on outcome. When a difference is established, a registry will undertake subsequent analyses in an attempt to identify factors that may or may not be associated with that difference. This is not attempting to assign causality, but to provide additional information that enables surgeons to effectively use the data to guide their choice of treatment options.

To optimize community outcomes of joint replacement surgery, it is not necessary to know why there is a difference. Incremental improvement can be achieved by surgeons choosing treatment options that have been identified as having better outcomes or alternatively avoiding those that have not. Those that attempt to rank the value of registry data with respect to the capacity to identify causality have entirely missed the point of the purpose of a registry and the approach it uses to achieve this.

This raises the question of how registry data should be valued. If it is felt important to rank against clinical trials, then criteria relevant to both should be used. Using new criteria that differ from the capacity to identify causality will significantly alter the perspective of relative value. Strong arguments could be made that registries have a greater capacity to provide new information, that the information they provide is more applicable, and that their ability to bring about beneficial clinical change is greater.

The predetermined limitations imposed by trial design, although necessary to establish causality, impede their ability to identify additional factors that may have the potential to influence outcome. This limitation does not occur with registry data analysis. Consequently, the potential for registry analysis to identify factors that have not previously been known to be associated with a particular outcome must be greater. The ability of a registry to do this is enhanced by the large numbers involved in registry analysis. Additionally, registries have the capacity to provide important information that could never be obtained through a clinical trial. This includes all community-based comparative outcomes, as well as providing insight into broad-based issues such as the impact of clinical experience or surgical skill.

In order to establish causality, a well-designed clinical trial must have high internal validity. This is achieved at the expense of external validity. An important effect of this is the subsequent limitation of the wider applicability of the trial results. A clinical trial may answer a specific question, but as a consequence of limited surgeon involvement and a restricted patient population, there may be difficulty in extrapolating these findings to community practice in order to achieve a beneficial outcome.

There are many other reasons why trials may have limited beneficial impact on the outcome of joint replacement in the community. These include, amongst others, non-availability of specific expertise and infrastructure used within the trial or a lack of relevance, particularly if the technology or approach has been superseded during the time it took to undertake the trial.

Stakeholder participation is another, often poorly considered difference. It has the potential to affect in a major way the ability to bring about beneficial change. In a clinical trial, stakeholders are limited to the surgeons, patients, and the prosthesis company involved. Registries, on the other hand, have very wide stakeholder involvement. Not only do they include all surgeons, patients, and companies but also hospitals and government within the community being surveyed. This greater participation results in broad-based data ownership. Change is more likely to occur if individual stakeholders are able to identify areas for potential improvement based on analysis of their own data. The implication of this is that a registry will have the most impact within the community contributing data. This may well be true, but it does not mean that the information provided by registries lacks relevance to other surgeons. On the contrary, registry reporting of community-based analysis has the utmost relevance to any individual surgeon’s practice. Wider access to registry reports is essential, and this is facilitated by publication in refereed journals. Using this approach to more widely disseminate registry analysis has the added benefit of ensuring the maintenance of reporting standards.

It is not clear what is to be gained by attempting to rank and contrast the value of information obtained from registries against clinical trials, particularly if the criteria used to rank that information is not relevant to registry analysis. Registries and clinical trials are two entirely separate approaches to data collection and analysis. It would seem that a more sensible approach would be to develop new criteria that can be used to assess and compare the value of registry data in its own right. Just like clinical trials, registries are individual—each with their own strengths and weaknesses. The type, completeness, and coverage of data collected by a registry, its stage of development, the strategies used for internal and external data validation, the approach to analysis, and—importantly—the issue being addressed by that analysis all contribute to the relative value of the data compared to other registry data.

Clinical trials and registries provide different information. One is not better than the other. They are complementary, and each has an important role in ensuring improved outcome of joint replacement. That outcome will be enhanced not only through a better understanding of the differences between clinical trials and registries, but also through an enhanced appreciation of the relevance and inherent value of the information provided by both.

